# New diarylmethanofullerene derivatives and their properties for organic thin-film solar cells

**DOI:** 10.3762/bjoc.5.7

**Published:** 2009-02-24

**Authors:** Daisuke Sukeguchi, Surya Prakash Singh, Mamidi Ramesh Reddy, Hideyuki Yoshiyama, Rakesh A Afre, Yasuhiko Hayashi, Hiroki Inukai, Tetsuo Soga, Shuichi Nakamura, Norio Shibata, Takeshi Toru

**Affiliations:** 1 Department of Frontier Materials, Graduate School of Engineering, Nagoya Institute of Technology, Gokiso, Showa-ku, Nagoya 466 8555, Japan

**Keywords:** bulk heterojunction solar cells, fullerene derivatives, high open-circuit voltage

## Abstract

A number of diarylmethanofullerene derivatives were synthesized. The cyclopropane ring of the derivatives has two aryl groups substituted with electron-withdrawing and -donating groups, the latter with long alkyl chains to improve solubility in organic solvents, an important property in processing cells. First reduction potentials of most derivatives were less negative than that of [6,6]-phenyl-C61-butyric acid methyl ester (PCBM), which is possibly ascribed to their electron-withdrawing nature. Organic thin-film photovoltaic cells fabricated with poly(3-hexylthiophene) (P3HT) as the electron-donor and diarylmethanofullerene derivatives as the electron-acceptor material were examined. The {(methoxycarbonyl)phenyl[bis(octyloxy)phenyl]methano}fullerene showed power conversion efficiency as high as PCBM, but had higher solubility in a variety of organic solvents than PCBM. The *V*_oc_ value was higher than that of PCBM, which is derived from the electron-donating (octyloxy)phenyl group, possibly raising the LUMO level. Photovoltaic effects of the devices fabricated with the derivatives having some electron-withdrawing groups were also examined.

## Introduction

Bulk heterojunction photovoltaic cells consisting of thin-film composites of conjugated polymers (donors) and fullerene derivatives (acceptors) are promising candidates for inexpensive, renewable solar energy conversion [1–6]. These organic thin films could also be used as flexible solar cells. Significant efforts have been made to optimize the power-conversion efficiency (PCE) of solar cells based on poly(3-hexylthiophene) (P3HT) as a donor and [6,6]-phenyl-C61-butyric acid methyl ester (PCBM) as an acceptor [7–10]. Using a combination of these donor and acceptor molecules together with the optimized solvent processing, composition, and effect of device annealing, PCE has reached up to 5% [11–17]. In addition to PCBM widely studied as the bulk heterojunction (BHJ) acceptor, several methanofullerene derivatives have so far been studied for the development of efficient acceptors such as spiroannulated methanofullerenes [18], diarylmethanofullerenes [19–20], alkoxy-substituted PCBM derivatives [21], and bisPCBM [22]. These derivatives have been shown to have excellent properties as acceptors, but it is still important to study the structure-efficiency relationship of the fullerene structure in order to develop highly efficient OPVs. The design of highly soluble derivatives of PCBM has also attracted much attention because 3D architectures such as vesicles and fibers are highly dependent on the solvent used [23–24]. Recently, we briefly communicated a soluble bulk-heterojunction organic photovoltaic P3HT device using a newly synthesized methanofullerene derivative **1a** [25]. In this paper, we give a full account of our studies on new bulk-heterojunction (BHJ) devices composed of P3HT and a variety of newly synthesized diarylmethanofullerene derivative **1a**–**k**, **2** blends.

## Results and Discussion

**Figure 1:**
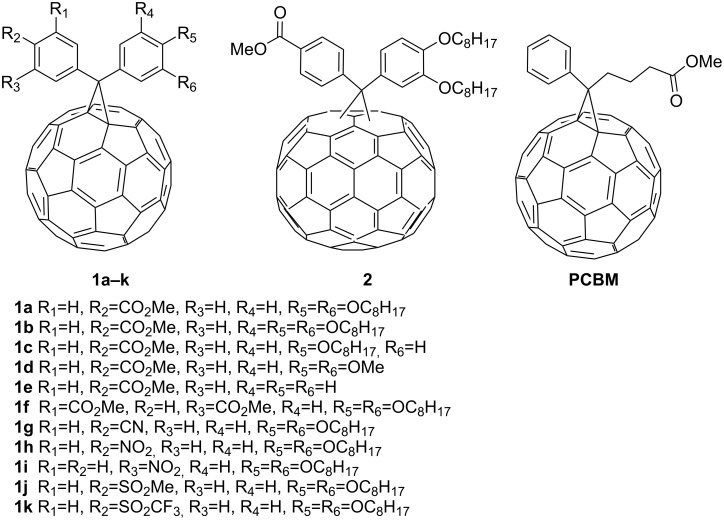
Diarylmethanofullerene derivatives.

We prepared new diarylmethanofullerene derivatives which were studied as electron-accepting materials in the bulk-heterojunction device of organic thin-film photovoltaic cells (Figure 1). The new diarylmethanofullerenes have two aromatic rings on the cyclopropane, having electron-withdrawing and electron-donating groups. The electron-withdrawing groups were expected to increase the electron-accepting ability of fullerene, while the electron-donating groups would decrease the LUMO energy level which leads to an increase of the open-circuit voltage (*V*_oc_) values of the solar cell. These diarylmethanofullerenes have long alkyl chain(s) to increase the solubility and hopefully to cause the interaction with the donor P3HT polymer, being related to the morphology of the heterojunction thin film.

### Synthesis of diarylmethanofullerene derivatives

**Scheme 1:**
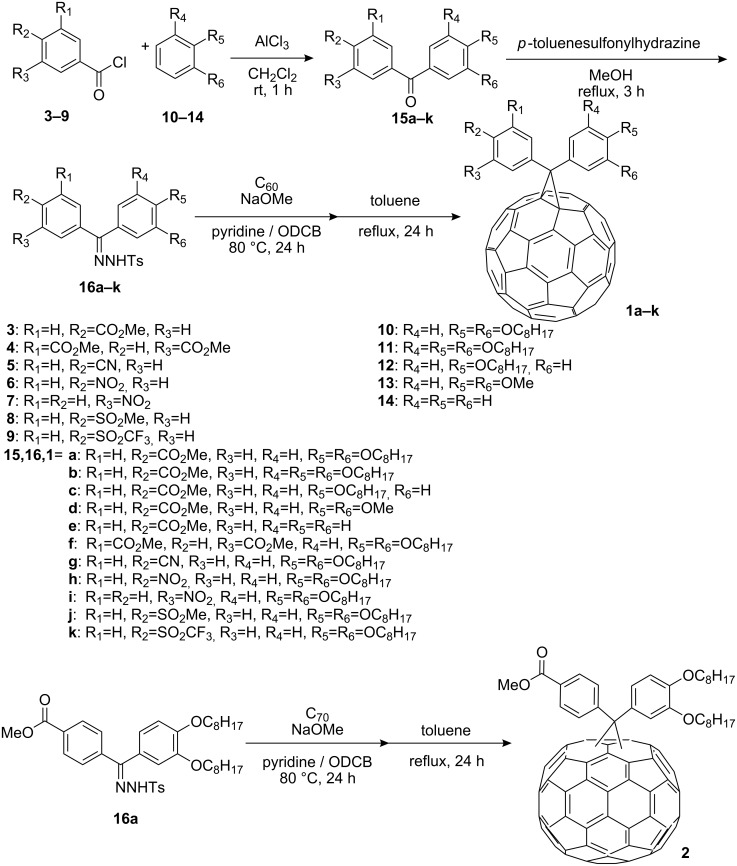
Synthesis of diarylmethanofullerene derivatives.

Diarylmethanofullerene derivatives were synthesized according to the method cited in the literature [26]. Synthetic routes are shown in Scheme 1.

Generally, the Friedel–Crafts acylation of benzene derivatives **10**–**14** with acid chlorides **3**–**9** in the presence of anhydrous AlCl_3_ gave the carbonyl compounds **15a**–**k** which were treated with *p*-toluenesulfonylhydrazine to afford the hydrazones **16a**–**k**. Treatment of **16a**–**k** with sodium methoxide or LiHMDS in *o*-dichlorobenzene (ODCB) gave a mixture of isomeric diarylmethanofullerenes. After purification, thermal treatment under reflux in toluene gave diarylmethanofullerenes **1a**–**k** in good yields. The C_70_ derivative **2**, a mixture of isomers, was prepared from **16a** by a procedure similar to those of C_60_ derivatives **1a**–**k** using C_70_ instead of C_60_. As expected, all the compounds were soluble in all organic solvents tested such as AcOEt, THF, acetone, dichloromethane, toluene and xylenes, while conventional PCBM has high solubility in toluene and xylenes, but limited solubility in AcOEt, THF, and acetone.

### Electrochemical Studies

Electrochemical studies were performed by differential pulse voltammetry (DPV) using a platinum electrode and tetrabutylammonium hexafluorophosphate as the supporting electrolyte in THF. Reduction potentials are listed in Table 1. The obtained cyclic voltammetry data were reversible. The first reduction potentials depend on the electron-donating nature of the substituents, in which tris(octyloxy)phenyl compound **1b** showed a more negative value than mono- and bis(octyloxy)phenylmethanofullerenes **1c** and **1a**. When compared with PCBM, most derivatives except **1e** showed first reduction potentials with less negative values. It is reasonable that less negative values are derived from the electron-withdrawing nature of the (methoxycarbonyl)phenyl, cyanophenyl, nitrophenyl, (methylsulfonyl)phenyl, and (trifluoromethylsulfonyl)phenyl groups attached to the cyclopropane ring, enough to compensate the opposite effect of the electron-donating mono-, bis-, or tris(octyloxy)phenyl group [19]. These first reduction potential data showed that the diarylmethanofullerene derivatives can be a better electron acceptor than PCBM [27].

**Table 1:** DPV Data for soluble fullerene derivatives in ODCB^a^

Derivative	E^1^_red_	E^2^_red_	E^3^_red_	E^4^_red_	E^5^_red_	E^6^_red_

**1a**	−0.900	−1.205	−1.420	−1.770	−2.270	
**1b**	−0.945	−1.376	−1.885	−2.100	−2.395	
**1c**	−0.905	−1.285	−1.505	−1.775	−2.265	
**1d**	−0.835	−1.210	−1.715	−2.155		
**1e**	−1.050	−1.320	−1.800	−2.275		
**1f**	−0.915	−1.290	−1.530	−1.705	−2.320	
**1g**	−0.905	−1.455	−1.836	−2.275		
**1h**	−0.890	−1.270	−1.770	−2.245		
**1i**	−0.890	−1.290	−1.590	−1.885	−2.170	−2.350
**1j**	−0.883	−1.253	−1.780	−2.252		
**1k**	−0.897	−1.279	−1.737	−1.873		
**2**	−0.915	−1.385	−1.565	−1.725	−1.865	−2.125
PCBM	−1.049	−1.290	−1.535	−1.785	−2.285	

^a^A 3 mm platinum plate was used as working electrode and a platinum wire as counter electrode. Ag/AgNO_3_ (0.01 M in MeCN/0.1 M TBAPF) was used as reference electrode separated by Vycor^®^ glass, and all potentials are given in relation to this electrode. The measurements were performed using a concentration of approximately 0.5 mM of the compounds.

### Photovoltaic Devices

**Figure 2:**
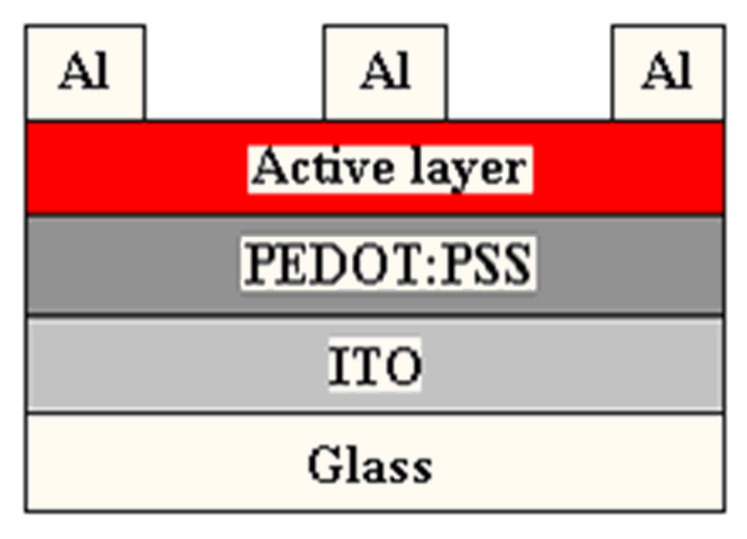
Device layout of the solar cell.

A series of solar cells was fabricated with P3HT as the electron-donor and diarylmethanofullerene derivatives as the electron-acceptor material. Sandwich configurations are described as ITO/PEDOT:PSS/P3HT:diarylmethanofullerene derivatives/Al (Figure 2). The ITO/PEDOT:PSS/P3HT:PCBM/Al cell was also fabricated for a comparative study. The conducting polymer, poly(3,4-ethylenedioxythiophene):poly(styrenesulfonate) (PEDOT:PSS), acting as a buffer layer for hole transport and reducing the roughness of the ITO surface, was spin-coated by 2000 rpm on the ITO-glass substrate with a thickness of ~80 nm from aqueous solution. The layer was then annealed at 120 °C for 10 min to remove the residual water. An ODCB solution of P3HT and the diarylmethanofullerene derivative or PCBM in a 1:1 weight ratio [28] prepared by stirring for a day was spin-coated and subsequently the device was heated at 130 °C for 30 min. The Al cathode layer was deposited with a thickness of ~100 nm by thermal evaporation in a vacuum chamber under 3 × 10^−4^ Pa. After thermal evaporation of the Al cathode layer, the device was annealed at 140 °C for 10 min in a glove box. The active area of the device was defined in an area of 3 mm^2^ with a shadow mask (Figure 2).

Current density–voltage (*J*–*V*) characteristics of the device were measured under one-sun illumination using an AM1.5G filter with a white light xenon lamp under a nitrogen atmosphere. A photovoltaic effect of these devices was clearly visible under exposure to light. OPVs fabricated with P3HT:diarylmethanofullerene derivatives having electron-withdrawing groups such as bis(methoxycarbonyl)phenyl, cyanophenyl, 3- and 4-nitrophenyl, (methylsulfonyl)phenyl, and (trifluoromethylsulfonyl)phenyl groups, taking a P3HT:PCBM-blended cell as a reference, were examined. Table 2 shows the current density–voltage (*J*–*V*) characteristic parameters of the devices structured from P3HT:diarylmethanofullerene derivatives **1a**–**1k**, **2** and P3HT:PCBM blends.

**Table 2:** *J*–*V* characteristic parameters of bulk–heterojunction solar cells

Derivative	*Eff* (%)^a^	*FF*^b^	*V*_oc_^c^	*J*_sc_^d^

**1a**	1.93	0.59	0.61	5.41
**1b**	1.50	0.49	0.66	4.60
**1c**	1.05	0.39	0.60	4.43
**1d**	0.022	0.18	0.49	0.25
**1e**	–^e^	–	–	–
**1f**	1.00	0.59	0.54	3.13
**1g**	0.84	0.40	0.52	4.09
**1h**	0.051	0.21	0.35	0.69
**1i**	–^e^	–	–	–
**1j**	1.24	0.50	0.56	4.43
**1k**	0.86	0.43	0.48	4.20
**2**	1.60	0.55	0.60	4.90
PCBM	1.98	0.56	0.52	6.82

^a^Power-conversion efficiency ^b^Fill factor ^c^Open-circuit voltage ^d^Open-circuit current ^e^Power conversion was not observed.

Derivative **1a** having methoxycarbonylphenyl and bis(octyloxy)phenyl groups was shown to be a superior acceptor in combination with P3HT and PCE of the P3HT:**1a** blended cell was as high as that of PCBM. Especially, the *V*_oc_ value of the P3HT:**1a** cell was 0.61 V, 0.09 V higher than that of the P3HT:PCBM cell. This could be generally ascribed to the reduced LUMO level of the methanofullerene by the electron-donating bis(octyloxy)phenyl group, since the band gap between the HOMO level of the P3HT and the LUMO level of the acceptor is related to the *V*_oc_ value, the higher band gap corresponding to the higher *V*_oc_ value [20,27,29–31]. More negative first reduction potentials of the methoxy-substituted PCBM derivatives in comparison with that of PCBM have been shown to be well correlated to the *V*_oc_ values of the solar cells [20]. On the other hand, the first reduction potential of **1a** is less negative than that of PCBM (Table 1). In addition, a higher *V*_oc_ value was shown for the P3HT-**1a** cell. The tris(octyloxy)phenyl- and (octyloxy)phenyl-substituted methanofullerenes **1b** and **1c** with less negative first reduction potential also showed enhanced *V*_oc_ values. The electron-donating (octyloxy)phenyl groups seemingly mainly worked on this effect. The first reduction potentials of the derivatives **1d**, **1f**–**1k**, and **2** having electron-withdrawing groups are also less negative than that of PCBM. It should be noted that the derivatives **1a**–**1c** having the methoxycarbonylphenyl group exhibited enhanced *V*_oc_ values as compared with lower *V*_oc_ values for the cells fabricated with other derivatives **1d**, **1f**–**1k**, and **2**. The reason is not clear but the lower *V*_oc_ values may be a consequence of negative effects such as morphology problems relating to inefficient charge transfer or charge separation. A more detailed study is necessary to elucidate these results. Short-circuit current (*J*_sc_) and fill factor (*FF*) values for **1a**–**1c** were lower in comparison with that for PCBM. Dimethoxyphenyl- and (methoxycarbonyl)phenyl-substituted, and phenyl- and (methoxycarbonyl)phenyl-substituted methanofullerenes **1d** and **1e** did not work as acceptors, probably because of their low solubility in ODCB. The bis(methoxycarbonyl)phenyl- and cyanophenyl-substituted methanofullerenes **1f** and **1g** showed good PCE, whereas nitrophenyl-substituted methanofullerenes **1h** and **1i** did not work as acceptors. (Methoxycarbonyl)phenylmethano-C_70_ derivative **2** showed excellent PCE. (Methylsulfonyl)phenyl- and (trifluoromethylsulfonyl)phenyl-substituted methanofullerenes **1j** and **1k** also worked fine.

**Figure 3:**
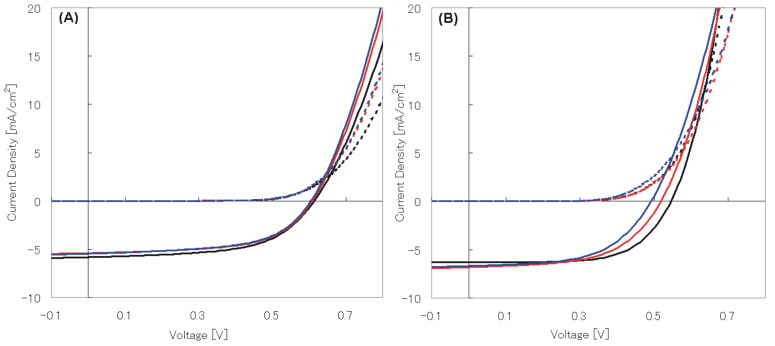
(A) *J*–*V* characteristics of the P3HT:**1a**-blended device annealed at 100–140 °C (black: 100 °C, red: 130 °C, blue: 140 °C, solid line: current under light, dashed line: current under dark). (B) *J*–*V* characteristics of the P3HT:PCBM-blended device annealed at 100–140 °C (black: 100 °C, red: 130 °C, blue: 140 °C, solid line: with light current, dashed line: with dark current).

**Figure 4:**
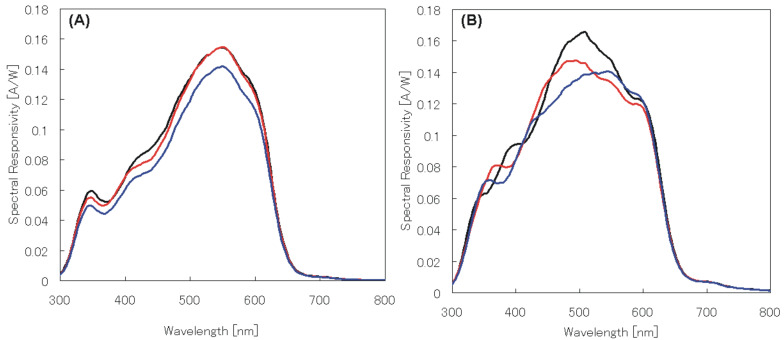
(A) Spectral responsivity of the P3HT:**1a**-blended film on the ITO glass annealed at 100–140 °C (100 °C: black, 130 °C: red, 140 °C: blue). (B) Spectral responsivity of the P3HT:PCBM-blended film on the ITO glass annealed at 100–140 °C (100 °C: black, 130 °C: red, 140 °C: blue).

The current density–voltage (*J*–*V*) characteristics of the OPVs structured from P3HT:**1a** and P3HT:PCBM blends were annealed at 140, 130, and 100 °C for 30 min after cathode (Al) deposition. The PCE values of the P3HT:**1a**-blended cell increased slightly as the annealing temperature decreased from 140 to 100 °C; thus, the PCE values of 1.91, 1.93, and 2.06% were obtained at the annealing temperatures of 140, 130, and 100 °C, respectively. Figure 3 shows the *J*–*V* characteristics of the P3HT:**1a** and P3HT:PCBM solar cells. Figure 4 shows the spectral responsibilities for both solar cells. *J*_sc_, *V*_oc_ and *FF* values of the P3HT:**1a**-blended cell did not significantly alter at these annealing temperatures [Figure 3(A)]. On the other hand, the *J*–*V* characteristics of the P3HT:PCBM-blended cell varied significantly depending on the annealing temperatures [Figure 3(B)].

**Figure 5:**
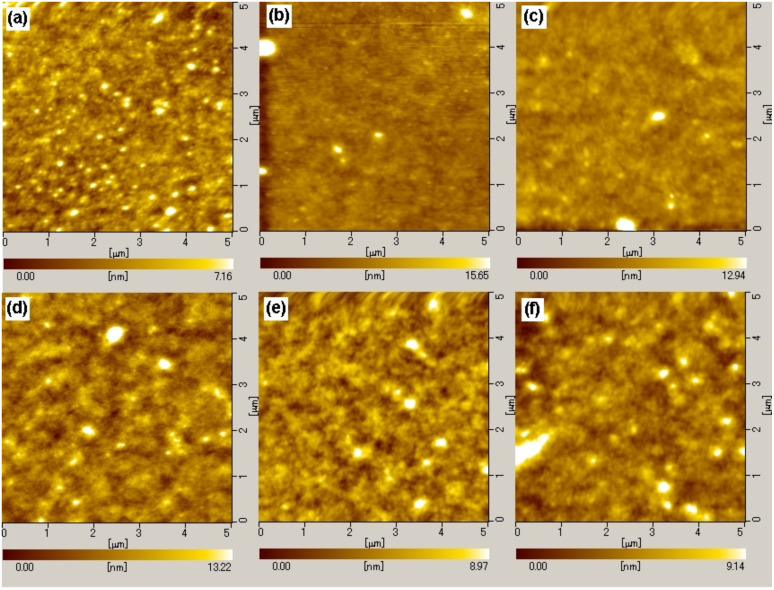
The AFM images of the P3HT:**1a**- and P3HT:PCBM-blended films annealed at different temperatures. (A) P3HT:**1a**-blended film annealed at 100 °C for 30 min, Ra:0.8161 nm, P-V: 15.02 nm: RMS: 1.110. (B) P3HT:**1a**-blended film annealed at 130 °C for 30 min Ra: 0.6775 nm, P-V: 17.51 nm, RMS: 0.9067 nm. (C) P3HT:**1a**-blended film annealed at 140 °C for 30 min, Ra: 0.8884 nm, P-V: 69.73 nm, RMS: 1.734 nm. (D) P3HT:PCBM-blended film annealed at 100 °C for 30 min, Ra: 1.531 nm, P-V: 35.72 nm, RMS: 2.159 nm. (E) P3HT:PCBM-blended film annealed at 130 °C for 30 min, Ra: 1.083 nm, P-V: 15.34 nm, RMS: 1.418 nm. (F) P3HT:PCBM-blended film annealed at 140 °C for 30 min, Ra: 1.135 nm, P-V: 13.47 nm, RMS: 1.573.

Figure 4(A) shows the spectral responsivities of the P3HT:**1a**-based cell with annealing at 140, 130, and 100 °C. Similar spectral responsivities were obtained irrespective of the annealing temperatures with slight decrease of that at 140 °C. The P3HT:**1a**-based cell showed a maximum at around 550 nm, whereas the P3HT:PCBM annealed at 100 and 130 °C exhibited the maxima at around 505 nm as shown in Figure 4(B). Since these responsivities are definitely derived from the absorption of P3HT, this notable bathochromic shift in the responsivity maxima of P3HT:**1a** in comparison with P3HT:PCBM can be ascribed to the change in the alignment of the P3HT polymer chain induced by the long octyloxy chains of **1a**. A change of the annealing temperature may also be related to the growth of the domain size of derivative **1a**, as observed in the case of PCBM [32–34], which possibly makes the conjugation length of P3HT longer. AFM measurements were performed on P3HT:**1a** and P3HT:PCBM films with different annealing temperatures. Different results were obtained between P3HT/**1a** and P3HT/PCBM: substantially similar low roughness values were observed in the AFM image of P3HT/**1a** annealed at 100, 130, 140 °C (r.m.s. = 0.9–1.1 nm). On the other hand, films of P3HT/PCBM showed similar roughness at 130 and 140 °C (r.m.s. = 1.4–1.6 nm), but a coarser surface was observed at 100 °C (r.m.s. = 2.2–2.6 nm). The P3HT/PCBM cell showed the highest efficiency. This is in accord with the data in which higher roughness gives a more efficient device [32–34]. As shown in Table 3, the highest efficiency was obtained for the P3HT:**1a** cell when annealed at 100 °C with a small PCE change in between 100 and 140 °C (Figure 5).

**Table 3:** *J*–*V* characteristic parameters of P3HT:**1a**- and P3HT:PCBM-blended solar cells at different annealing temperatures

Cell	Temperature (°C)	*Eff* (%)	*FF*	*V*_oc_	*J*_sc_

P3HT:**1a**	60	1.93	0.54	0.55	6.41
	80	1.88	0.56	0.57	5.80
	100	2.06	0.57	0.61	5.83
	130	1.93	0.59	0.61	5.41
	140	1.91	0.58	0.61	5.47

P3HT:PCBM	100	2.19	0.64	0.55	6.31
	130	1.98	0.56	0.52	6.82
	140	1.84	0.56	0.49	6.69

## Conclusion

In conclusion, a variety of new and highly soluble diarylmethanofullerene derivatives **1a**–**k** having electron-withdrawing and -donating groups were studied as acceptors of the BHJ device. Compared with the P3HT:PCBM blend, the P3HT:diarylmethanofullerene derivatives blend had decreased first reduction potential due to the electron-withdrawing aryl groups attached to the cyclopropane ring, possibly enhancing the acceptability of the fullerene moiety. Higher *V*_oc_ values were obtained due mainly to the electron-donating group, while the *J*_sc_ values were lowered. In particular, derivative **1a** having (methoxycarbonyl)phenyl and bis(octyloxy)phenyl groups was shown to be a superior acceptor in combination with P3HT and the PCE of the P3HT:**1a** blended cell was as high as that of PCBM. The annealing conditions were shown to be important in further improvement of the PCE values. The highest PCEs of both cells were obtained at a lower annealing temperature (100 °C). Their spectral responsivities and the AFM measurements suggested a change of the P3HT alignment depending on the annealing temperatures. These observations will lead the way to further development of highly efficient acceptors for organic thin-film solar cells.

## Experimental

### Representative procedure: Preparation of **1a**

#### Methyl 4-[3,4-bis(octyloxy)benzoyl]benzoate (**15a**)

To a solution of terephthalic acid monomethyl ester chloride (**3**, 268 mg, 1.35 mmol) in CH_2_Cl_2_ (6 mL) was added aluminium trichloride (199 mg, 1.49 mmol) in portions at 0 °C and subsequently a solution of 1,2-bis(octyloxy)benzene (**10**, 500 mg, 1.49 mmol) in CH_2_Cl_2_ (4 mL). The reaction mixture was stirred for 45 min. The resultant clear solution was stirred for an additional 20 min at 40 °C, when the color of the reaction mixture turned reddish brown. The reaction mixture was washed with brine. The organic layer was dried over MgSO_4_ and evaporated under vacuum to leave a solid which was purified by silica gel column chromatography (ethyl acetate/hexane = 10:90 as eluent) to give **15a** (624 mg, 93%). ^1^H NMR (CDCl_3_, 200 MHz): δ 8.14 (d, *J* = 7.8 Hz, 2H), 7.79 (d, *J* = 8.0 Hz, 2H), 7.46 (s, 1H), 7.32 (dd, *J* = 2.0, 13.6 Hz, 1H), 6.88 (d, *J* = 8.4 Hz, 1H), 4.10–4.00 (m, 4H), 3.96 (s, 3H), 1.89–1.80 (m, 4H), 1.56–1.28 (m, 20H), 0.91 (t, *J* = 6.6 Hz, 6H); ^13^C (CDCl_3_, 50.3 MHz): δ 194.32, 166.00, 153.33, 148.59, 142.01, 132.33, 129.14, 129.09, 129.03, 125.36, 113.85, 111.13, 69.21, 69.04, 52.42, 31.91, 29.47, 29.44, 29.37, 29.24, 29.14, 26.12, 26.09, 22.81, 14.27; FT-IR (KBr, cm^−1^): 2954, 2929, 2856, 1725, 1642; MS (EI) *m*/*z* 496; Anal. calcd. for C_31_H_44_O_5_: C, 74.96; H, 8.93. Found: C; 75.03, H; 8.92.

#### Methyl 4-{1-(2-tosylhydrazono)-1-[3,4-bis(octyloxy)phenyl]methyl}benzoate (**16a**)

A solution of **15a** (1 g, 2.01 mmol) and *p*-toluenesulfonylhydrazine (937 mg, 5.03 mmol) in methanol (15 mL) was heated under reflux for 5 h. The solvent was removed under vacuum to give a solid which was purified by silica gel column chromatography (ethyl acetate/hexane = 25:75 as eluent) to give **16a** (800 mg, 63%). ^1^H NMR (CDCl_3_, 200 MHz): δ 8.17 (d, *J* = 8.2 Hz, 2H), 7.85 (d, *J* = 8.2 Hz, 2H), 7.32–7.17 (m, 5H), 6.65–6.61 (m, 2H), 3.98–3.90 (m, 4H, s, 3H), 2.43 (s, 3H), 1.85–1.58 (m, 4H), 1.57–1.29 (m, 20H), 0.90 (t, *J* = 6.4 Hz, 6H); ^13^C (CDCl_3_, 50.3 MHz): δ 165.63, 153.10, 150.78, 148.44, 143.79, 135.69, 135.05, 131.17, 130.37, 129.28, 129.02, 128.36, 128.29, 127.7, 125.94, 121.51, 111.85, 111.37, 69.05, 68.91, 52.46, 31.89, 31.85, 29.48, 29.38, 29.31, 29.27, 29.15, 29.16, 26.05, 22.78, 22.75, 21.73, 14.23; FT-IR (KBr, cm^−1^): 2928, 2854, 1725, 1509; MS (EI) *m*/*z* 664; Anal. calcd. for C_38_H_52_N_2_O_6_S: C; 68.64, H; 7.88, N; 4.21. Found: C; 68.92, H; 8.19, N; 4.21.

#### Methyl 4-{[6,6]-1-[3,4-bis(octyloxy)phenyl]C61}benzoate (**1a**)

To a solution of **16a** (613 mg, 0.92 mmol) in dry pyridine (5.0 mL) was added sodium methoxide (50 mg, 0.92 mmol). After stirring for 30 min at room temperature, a solution of C_60_ (398 mg, 0.55 mmol) of in dry ODCB (10 mL) was added. The resulting mixture was stirred at 80 °C for 24 h. The reaction mixture was washed with diluted HCl aqueous solution and brine successively. Solvents were removed under vacuum to give the crude product which was purified by column chromatography (silica gel; toluene). First fraction was unreacted C_60_. Fractions containing brown color were collected and the solvent was evaporated to leave a solid. This compound was dissolved in toluene and refluxed for 24 h to complete the isomerisation. The isomerisation was confirmed by HPLC (Develosil ODS-HG-5, MeOH/CHCl_3_) and ^13^C NMR. Further purification by column chromatography as above yielded **1a** (112 mg, 17%). ^1^H NMR (CDCl_3_, 200 MHz): δ 8.15 (br s, 4H), 7.61 (m, 2H), 6.96 (d, *J* = 8.0 Hz, 2H), 4.12–3.98 (m, 4H), 3.92 (s, 3H), 1.83–1.79 (m, 4H), 1.56–1.28 (m, 20H), 0.87 (br s, 6H); ^13^C (CDCl_3_, 50.3 MHz) δ 166.23, 149.18, 148.30, 147.60, 147.54, 144.99, 144.82, 144.42, 144.36, 144.33, 143.97, 143.50, 142.62, 142.57, 141.94, 141.88, 141.80, 140.58, 138.12, 137.67, 130.60, 130.04, 129.85, 129.51, 123.95, 117.10, 113.05, 78.78, 69.81, 68.98, 57.46, 52.31, 32.00, 31.96, 29.65, 29.54, 29.44, 26.26, 26.22, 22.86, 14.38; FT-IR (KBr, cm^−1^): 2923, 2853, 1725, 1608, 1511, 1464, 1430, 1275, 1186, 1136, 1107; MALDI-TOF MS: *m/z* 1200; Anal. calcd. for C_91_H_44_O_4_: C; 90.98, H; 3.69. Found: C; 89.41, H; 3.45.

### Photovoltaic cells

After rubbing with cloth to remove the protrusions, the ITO-glass (FINE brand, Furuuchi Co. Ltd., 15 Ω/cm^2^) substrate was cleaned successively with acetone, methanol, and a copious amount of water, and dried by a N_2_ blower. The conducting polymer, poly(3,4-ethylenedioxythiophene):poly(styrenesulfonate) (PEDOT:PSS, Sigma-Aldrich), acting as a buffer layer for hole transport and reducing the roughness of the ITO surface, was spin-coated by 2000 rpm on the ITO-glass substrate with a thickness of ~80 nm from the aqueous solution. These layers were then annealed at 100 °C for 10 min to remove the residual water. P3HT was purchased from Sigma-Aldrich. An ODCB solution (15 mg/mL) of P3HT and the diarylmethanofullerene derivatives **1a**–**k**, **2** in a 1:1 weight ratio prepared by stirring for a day was spin-coated by 2000 rpm and the resultant layers were heated at appropriate temperatures. The Al cathode layer was deposited on the film with a thickness of ~100 nm by thermal evaporation in a vacuum chamber under 3 × 10^−4^ Pa. The active area of the device was defined in an area of 3 mm^2^ with a shadow mask. After thermal evaporation of the Al cathode layer, the device was annealed at 140 °C for 10 min. All the above operations were conducted in a glove box filled with nitrogen. Current density–voltage (*J*–*V*) characteristics of the device was measured by a JASCO instrument under one-sun illumination using an AM 1.5G filter with a white light xenon lamp (100 mW/cm^2^) under the nitrogen atmosphere. Optical absorption spectra of the device were measured by UV/VIS/NIR spectrophotometer (V-570 JASCO). The device with P3HT:PCBM was also fabricated for a comparative study and characterized as in the case of the P3HT:diarylmethanofullerene derivatives device.

## Supporting Information

Experimental procedures for the preparation of derivatives **1b**–**k**, **2**.

File 1 Experimental methods
