# Supplementary material for: New diarylmethanofullerene derivatives and their properties for organic thin-film solar cells
Source: Beilstein J Org Chem. 2009 Feb 24;5:7. doi: 10.3762/bjoc.5.7 (PMC2693951; doi:10.3762/bjoc.5.7)
Supplement: File 1 — Experimental methods [file Beilstein_J_Org_Chem-05-07-s001.pdf]

Supporting Information (Experimental)

New diarylmethanofullerene derivatives and their properties for organic thin-film solar cells

Daisuke Sukeguchi, Surya Prakash Singh, Mamidi Ramesh Reddy, Hideyuki Yoshiyama, Rakesh A. Afre, Yasuhiko Hayashi, Hiroki Inukai, Tetsuo Soga, Shuichi Nakamura, Norio Shibata* and Takeshi Toru

Address: Department of Frontier Materials, Graduate School of Engineering, Nagoya Institute of Technology, Gokiso, Showa-ku, Nagoya 466 8555, Japan

Experimental

General

All solvents were dried and distilled according to standard procedures. All of the reactions were monitored by thin-layer chromatography (TLC) carried out on 0.25 mm Merck silica-gel plate (60F-254). Column chromatography was carried out on a column packed with silica gel 60N spherical neutral size 63–210 μm. ^{1}H NMR (200 MHz) and ^{13}C (50.3 MHz) spectra were taken on a Varian Gemini-200 spectrometer. Chemical shifts are expressed in ppm downfield from tetramethylsilane. Infrared (IR), UV-vis spectra were recorded on a JASCO FT/IR-200 Spectrometer, V-530 spectrometer. Mass spectra of MALDI-TOF were taken on a SHIMADZU Axima CFR plus. Reverse phase HPLC analyses were performed on a JASCO PU-2080 Plus using 4.6 x 250 mm Develosil ODS-HG-5 column and MD-2015 multiwavelength detector. Electrochemical experiments were carried out on a BAS 50W electrochemical analyzer (Bioanalytical Systems, Inc., West Lafayette, IN 47906, USA). Tetrabutylammonium hexafluorophosphate (TBAPF) was purchased from TCI and recrystallized from EtOH. The solvent THF (Kanto Chemical Co. Inc., tetrahydrofuran, dehydrated stabilizer free) was passed through Glass Contour Ultimate Solvent System. For CV and DPV, a 3 mm platinum plate was used as working electrode and platinum wire as counter electrode. Ag/$AgNO_3$ (0.01 M in MeCN/0.1 M TBAPF) was used as reference electrode separated by a vycor glass, and all potentials given relate to this electrode. The measurements were performed using a concentration of approximately 0.5 mM of the compounds. The AFM measurements were taken on a Seiko SPI3800N.

Synthesis

Methyl 4-[3,4,5-tris(octyloxy)benzoyl]benzoate (15b)

Treatment as in the procedure of **15a** using **3** (1.54 g, 7.76 mmol), aluminium trichloride (1.15 g, 8.62 mmol) and 1,2,3-tris(octyloxy)benzene (**11**, 3.99 g, 8.62 mmol) gave **15b** (2.4 g, 50%). ^{1}H NMR ($CDCl_3$, 200 MHz): δ 8.15 (d, J = 8.4 Hz, 2H), 7.69 (d, J = 8.4 Hz, 2H), 7.22 (d, J = 8.8 Hz, 1H), 6.44 (d, J = 9.2 Hz, 1H), 4.08–4.0 (m, 6H), 3.96 (s, 3H), 1.87–1.73 (m, 6H), 1.57–1.28 (m, 30H), 0.91 (t, J = 6.2 Hz, 9H); ^{13}C ($CDCl_3$, 50.3 MHz): δ 198.98, 165.77, 158.52, 157.86,

141.77, 135.77, 132.15, 129.42, 129.15, 128.43, 113.85, 103.63, 68.88, 52.82, 31.96, 31.88, 30.32, 29.59, 29.43, 29.39, 29.34, 29.21, 26.10, 22.79, 14.23; FT-IR (KBr, cm^{-1}):2919, 2873, 2850, 1727, 1683, 1625, 1586, 1499, 1468, 1438, 1350, 1282, 1206, 1105; MS (EI) *m*/*z* 624; Anal. calc. for $C_{39}H_{60}O_6$: C; 74.96, H; 9.68. Found: C; 74.85, H; 9.37.

Methyl 4-[4-(octyloxy)benzoyl]benzoate (15c)

Treatment as in the procedure of **15a** using **3** (4.33 g, 21.81 mmol), aluminium trichloride (3.23 g, 24.2 mmol) and octyloxybenzene (**12**, 5.0 g, 24.23 mmol) gave **15c** (6.0 g, 75%). ^{1}H NMR ($CDCl_3$, 200 MHz): δ 8.14 (d, *J* = 8.2 Hz, 2H), 7.81–7.75 (m, 4H), 6.96 (d, *J* = 8.4 Hz, 2H), 4.06 (t, *J*= 6.6 Hz, 2H), 3.95 (s, 3H), 1.85–1.78 (m, 2H), 1.46–1.29 (m, 10H), 0.92 (t, *J* = 6.6 Hz, 3H); ^{13}C NMR ($CDCl_3$, 50.3MHz): δ 194.18, 165.96, 162.88, 141.92, 132.42, 132.32, 129.40, 129.02, 113.98, 68.32, 52.41, 31.90, 29.43, 29.32, 29.20, 26.11, 22.78, 14.26; FT-IR (KBr, cm^{-1}): 2955, 2923, 1721, 1604, 1572, 1507; EI-MS: *m/z* 368; Anal. Calcd for $C_{23}H_{28}O_4$: C; 74.97, H; 7.66. Found: C; 74.44, H; 7.94.

Methyl 4-(3,4-dimethoxybenzoyl)benzoate (15d)

Treatment as in the procedure of **15a** using **3** (1.80 g, 9.12 mmol), aluminium trichloride (1.35 g, 10.1 mmol) and 1,2-dimethoxybenzene (**13**, 1.40 g, 10.1 mmol) gave **15d** (2.0 g, 73%). ^{1}H NMR ($CDCl_3$, 200 MHz): δ 8.15 (d, *J* = 8.0 Hz, 2H), 7.80 (d, *J* = 8.2 Hz, 2H), 7.49 (d, *J* = 2.0 Hz, 1H), 7.36 (dd, *J* = 2.2, 8.6 Hz, 1H), 6.90 (d, *J* = 8.2 Hz, 1H), 3.96 (d, *J* = 4.2 Hz, 9H); ^{13}C NMR ($CDCl_3$, 50.3MHz): δ 194.20, 165.92, 153.08, 148.80, 141.82, 132.44, 129.33, 129.11, 125.42, 111.68, 109.63, 56.12, 56.03, 52.42; FT-IR (KBr, cm^{-1}): 1725, 1647; EI-MS: *m/z* 300; Anal. calcd. for $C_{17}H_{16}O_5$: C; 67.99, H; 5.37. Found: C; 66.35, H; 5.63.

Methyl 4-benzoylbenzoate (15e)

Treatment as in the procedure of **15a** using **3** (5.00 g, 25.2 mmol) and aluminum trichloride (3.36 g, 25.17 mmol) in benzene (**14**) gave **15e** (4.2 g, 69%). ^{1}H NMR ($CDCl_3$, 200 MHz): δ 8.15–8.11 (m, 2H), 7.84–7.76 (m, 4H), 7.60–7.44 (m, 3H), 3.96 (s, 3H); ^{13}C NMR ($CDCl_3$, 50.3 MHz): δ 194.10, 165.75, 164.63, 164.53, 139.85, 135.45, 133.65, 132.20, 131.83, 131.71, 129.20, 128.64, 128.37, 128.31, 128.14, 128.05, 127.23, 51.41; FT-IR (KBr, cm^{-1}): 1718, 1646; EI-MS: *m/z* 240; Anal. calcd. for $C_{15}H_{12}O_3$: C; 74.99, H; 5.03. Found: C; 74.35, H; 4.97.

Dimethyl 5-[3,4-bis(octyloxy)benzoyl]benzene-1,3-dioate (15f)

Treatment as in the procedure of **15a** using 3,5-bis(methoxycarbonyl)benzoyl chloride (**4**, 1.17 g, 4.57 mmol), aluminum trichloride (678 mg, 5.08 mmol) and 1,2-bis(octyloxy)benzene (**10**, 1.70 g, 5.08 mmol) gave **15f** (2.1 g, 83%). ^{1}H NMR ($CDCl_3$, 200 MHz): δ 8.95–8.93 (m, 1H), 8.90 (d, *J* = 1.8 Hz, 1H), 8.52 (m, 1H), 8.56 (d, *J* = 1.8 Hz, 1H), 7.46 (d, *J* = 2.0 Hz, 1H), 7.29 (d, *J* = 2.0 Hz, 1H), 6.89 (d, *J* = 8.4 Hz, 1H), 4.10–4.0 (m, 4H), 3.96 (s, 3H), 1.89–1.80 (m, 4H), 1.56–1.28 (m, 20H), 0.91 (t, *J* = 6.6 Hz, 6H); ^{13}C NMR ($CDCl_3$, 50.3 MHz): δ 192.99, 165.11, 164.93, 164.23, 153.50, 148.75, 139.01, 136.16, 135.40, 135.01, 134.70, 134.24, 134.09, 132.98, 131.61, 130.90, 130.56, 128.67, 125.29, 113.78, 111.27, 69.23, 69.05, 52.89, 52.60, 31.89, 29.42, 29.36, 29.21, 29.12, 26.11, 26.09, 22.78, 14.23; FT-IR (KBr, cm^{-1}): 2924, 2854, 1732, 1640, 1447, 1430, 1394, 1339, 1254; EI-MS: *m/z* 554; Anal. calcd. for $C_{33}H_{46}O_7$: C; 71.45, H; 8.36. Found: C; 71.70, H; 8.24.

4-[3,4-Bis(octyloxy)benzoyl]benzonitrile (15g)

Treatment as in the procedure of **15a** using 4-cyanobenzoyl chloride (**5**, 3.12 g, 18.8 mmol), aluminium trichloride (2.79 g, 20.9 mmol)and 1,2-bis(octyloxy)benzene (**10**, 7.00 g, 20.9 mmol) yielded **15g** (6.77 g, 78%). ^{1}H NMR ($CDCl_3$, 200 MHz): δ 7.83 (m, 4H), 7.44 (s, 1H), 7.27 (dd, *J* = 2.0, 4.6 Hz, 1H), 6.87 (d, *J* = 8.4, 1H), 4.10–4.0 (m, 4H), 1.90–1.80 (m, 4H), 1.58–1.28 (m, 20H), 0.91 (t, *J* = 6.8 Hz, 6H); ^{13}C NMR ($CDCl_3$, 50.3 MHz): δ 193.24, 153.63, 148.74, 141.95, 131.71, 129.60, 128.41, 125.41, 117.89, 114.76, 113.60, 111.07, 69.21, 69.05, 31.89, 29.44, 29.41, 29.35, 29.21, 29.09, 26.10, 26.07, 22.79, 14.26; FT-IR (KBr, cm^{-1}): 2952, 2924, 2869, 2852, 2229, 1652, 1590, 1577, 1521, 1466, 1436, 1420, 1392, 1348, 1310, 1301, 1282, 1224, 1178, 1149; Anal. calcd. for $C_{30}H_{41}NO_3$: C; 77.71, H; 8.91, N; 3.02. Found: C; 77.67, H; 9.24, N; 2.95.

1-[3,4-Bis(octyloxy)phenyl]-1-(4-nitrophenyl)methanone (15h)

Treatment as in the procedure of **15a** using 4-nitrobenzoyl chloride (**6**, 4.99 g, 26.9 mmol), aluminum trichloride (3.98 g, 29.89 mmol) and 1,2-bis(octyloxy)benzene (**10**, 10.0 g, 29.89 mmol) gave **15h** (11.3 g, 87%). ^{1}H NMR ($CDCl_3$, 200 MHz): δ 8.33 (d, *J* = 10.0 Hz, 2H), 7.89 (d, *J* = 6.6 Hz, 2H), 7.46 (s, 1H), 7.28 (dd, *J* = 2.2, 8.0 Hz, 1H), 6.88 (d, *J* = 8.4 Hz, 1H), 4.10–4.0 (m, 4H), 1.90–1.81 (m, 4H), 1.55–1.28 (m, 20H), 0.91 (t, *J* = 6.2 Hz, 6H); ^{13}C NMR ($CDCl_3$,

50.3 MHz): δ 192.99, 153.75, 149.04, 148.78, 143.66, 130.00, 128.38, 125.49, 123.11, 113.53, 111.08, 69.22, 69.06, 31.90, 29.46, 29.43, 29.37, 29.22, 29.10, 26.12, 26.08, 22.80, 14.26; FT-IR (KBr, cm^{-1}): 2926, 2853, 1654, 1588, 1524, 1466; MS (EI) *m/z* 483; Anal. calcd. for $C_{29}H_{41}NO_5$: C; 72.02, H; 8.54, N; 2.90. Found: C; 71.92, H; 8.27, N; 2.82.

(3-Nitrophenyl)[3,4-bis(octyloxy)phenyl]methanone (15i)

Treatment as in the procedure of **15a** using 3-nitrobenzoyl chloride (**7**, 4.99 g, 26.90 mmol), aluminum trichloride (3.98 g, 29.89 mmol) and 1,2-bis(octyloxy)benzene (**10**, 10 g, 29.89 mmol) gave **15i** (10.5 g, 81%). ^{1}H NMR ($CDCl_3$, 200 MHz): δ 8.56 (s, 1H), 8.42 (d, *J* = 8.4 Hz, 1H), 8.09 (d, *J* = 7.6 Hz, 1H), 7.71–7.63 (m, 1H), 7.46 (s, 1H), 7.30 (m, 2H), 6.90 (d, *J* = 8.6 Hz, 1H), 4.11–4.02 (m, 4H), 1.87–1.81 (m, 4H), 1.58–1.29 (m, 20H), 0.88 (t, *J* = 6.0 Hz, 6H); ^{13}C NMR ($CDCl_3$, 50.3 MHz): δ 192.30, 153.69, 148.83, 147.56, 145.64, 145.49, 139.60, 134.85, 129.13, 128.33, 125.79, 125.29, 124.11, 120.97, 119.76, 114.19, 113.76, 111.40, 111.23, 69.26, 69.07, 68.82, 31.88, 29.43, 29.35, 29.23, 29.12, 26.09, 22.78, 14.23; FT-IR (KBr, cm^{-1})**:** 2954, 2925, 2853, 1645, 1577, 1536, 1515, 1503; EI-MS: *m/z* 483; Anal. calcd. for $C_{29}H_{41}NO_5$: C; 72.02 H; 8.54 N; 2.90. Found: C; 72.04, H; 8.44, N; 2.82.

1-[4-(Methylsulfonyl)phenyl]-1-[3,4-bis(octyloxy)phenyl]methanone (15j)

Treatment as in the procedure of **15a** using 4-methylsulfonylbenzoyl chloride (**8**, 3.01 g, 9.00 mmol), aluminum trichloride (1.19 g, 9.00 mmol) and 1,2-bis(octyloxy)benzene (**10**, 1.64 g, 7.50 mmol) yielded **15j** (1.41 g, 30%). ^{1}H NMR ($CDCl_3$, 200 MHz): δ 0.92–0.85 (m, 6H), 1.52–1.29 (m, 20H), 1.91–1.79 (m, 4H), 3.10 (s, 3H), 4.11–4.01 (t, *J* = 6.6 Hz, 4H), 6.86 (d, *J* = 8.4 Hz, 1H), 7.29–7.25 (m, 1H), 7.47–7.46 (m, 1H), 7.88–7.86 (m, 2H), 8.07–8.02 (m, 2H); FT-IR (KBr, cm^{-1}): 3056, 3023, 2926, 1749.

1-[3,4-Bis(octyloxy)phenyl]-1-(4-trifluoromethylsulfonylphenyl)methanone (15k)

Treatment as in the procedure of **15a** using 4-(trifluoromethylsulfonyl)benzoyl chloride (**9**, 4.22 g, 15.46 mmol), aluminum trichloride (3.06 g, 23.2 mmol) and 1,2-bis(octyloxy)benzene (**10**, 7.58 g, 23.2 mmol) gave **15k** (3.04 g, 35%). ^{1}H NMR ($CDCl_3$, 200 MHz): δ 8.14 (d, *J* = 8.0 Hz, 2H), 7.94 (d, *J* = 8.2 Hz, 2H), 7.48–7.47 (m, 1H), 7.26–7.25 (m, 1H), 6.86 (d, *J* = 8.4 Hz, 1H),

4.11–3.95 (m, 4H), 1.98–1.79 (m, 4H), 1.56–1.16 (m, 20H), 0.88–0.79 (m, 6H); ^{19}F NMR ($CDCl_3$, 188 MHz) δ 77.78, 77.85; FT-IR (KBr, cm^{-1}): 3093, 3031, 2957, 1751.

1-[(4-Methoxycarbonyl)phenyl]-1-[3,4,5-tris(octyloxy)phenyl]methanone *p*-tolylsulfonylhydrazone (16b)

Treatment as in the procedure of **16a** using **15b** (2.29 g, 3.66 mmol) and *p*-tosylhydrazine (2.04 g, 11.0 mmol) gave **16b** (1.8 g, 63%). ^{1}H NMR ($CDCl_3$, 200 MHz): δ 8.20 (d, *J* = 8.4 Hz, 2H), 7.81 (d, *J* = 8.4 Hz, 2H), 7.35(d, *J* = 8.4 Hz, 2H), 7.18 (d, *J* = 8.2 Hz, 2H), 6.2 (s, 2H), 4.05–3.88 (m, 6H, s, 3H), 2.43 (s, 3H), 1.84–1.74 (m, 6H), 1.59–1.30 (m, 30H), 0.90 (t, *J* = 6.2 Hz, 9H). FT-IR (KBr, cm^{-1}): 3202, 2925, 2855, 1722, 1707, 1618, 1514, 1411, 1292, 1168, 1104, 1077; EI-MS *m/z* 792; Anal. calcd. for $C_{46}H_{68}N_2O_7S$: C; 69.66, H; 8.64, N; 3.53. Found: C; 69.55, H; 8.23, N; 3.64.

1-[(4-Methoxycarbonyl)phenyl]-1-[4-(octyloxy)phenyl]methanone *p*-tolylsulfonylhydrazone (16c)

Treatment as in the procedure of **16a** using **15c** (2.00 g, 5.43 mmol) and *p*-tosylhydrazine (3.00 g, 16.3 mmol) gave **16c** (2.2 g, 76%). ^{1}H NMR ($CDCl_3$, 200 MHz): δ 7.94–7.82 (m, 4H), 7.74 (s, 1H), 7.50 (d, *J* = 8.4 Hz, 2H), 7.34 (d, *J* =8.6 Hz, 2H) 7.02 (m, 3H), 4.03 (t, *J* = 6.6 Hz, 2H), 3.89 (s, 3H), 2.42 (s, 3H), 1.82–1.78 (m, 2H), 1.32–1.29 (m, 10H), 0.92 (t, *J* = 6.6 Hz, 3H); ^{13}C NMR ($CDCl_3$, 50.3 MHz): δ 166.22, 160.19, 152.74, 143.96, 140.73, 135.20, 130.65, 129.63, 129.45, 129.14, 127.69, 127.34, 121.73, 115.55, 68.26, 52.25, 31.95, 29.46, 29.39, 29.28, 26.19, 22.83, 21.80, 14.30; FT-IR (KBr, cm^{-1}): 3192, 2931, 2850, 1723, 1639; EI-MS: *m/z* 536; Anal. calcd. for $C_{30}H_{36}N_2O_5S$: C; 67.14, H; 6.76, N; 5.52. Found: C; 67.10, H; 6.77, N; 5.32.

1-[4-(Methoxycarbonyl)phenyl]-1-(3,4-dimethoxyphenyl)methanone *p*-tolylsulfonylhydrazone (16d)

Treatment as in the procedure of **16a** using **15d** (1.7 g, 5.66 mmol) and *p*-tosylhydrazine (3.16 g, 16.98 mmol) gave **16d** (1.80 g, 68%). ^{1}H NMR ($CDCl_3$, 200 MHz): δ 7.95–7.81 (m, 4H), 7.78 (d, *J* = 10.6 Hz, 2H), 7.35–7.18 (m, 2H), 6.99 (d, *J* = 8.2 Hz, 1H), 6.85 (d, *J* = 5.0 Hz, 1H) 6.66 (d, *J* = 8.2 Hz, 1H), 3.96–3.82 (m, 9H), 2.43 (s, 3H); EI-MS: *m/z* 468; Anal. calcd. for $C_{24}H_{24}N_2O_6S$: C; 61.52, H; 5.16, N; 5.98. Found: C; 61.32, H; 5.10, N; 5.97.

1-[4-(Methoxycarbonyl)phenyl]-1-phenylmethanone *p*-tolylsulfonylhydrazone (16e): Treatment as in the procedure of **16a** using **15e** (1.00 g, 4.16 mmol) and *p*-tosylhydrazide (2.3 mg, 12.5 mmol) gave **16e** (1.30 g, 77%). ^{1}H NMR ($CDCl_3$, 200 MHz): δ 8.38 (d, *J* = 5.6 Hz, 1H), 8.09 (d, *J* = 8.2 Hz, 2H), 7.83–7.67 (m, 5H), 7.53 (d, *J* = 6.4 Hz, 1H), 7.32–7.26 (m, 4H), 3.93 (s, 3H), 2.43 (s, 3H); FT-IR (KBr, cm^{-1}): 1721; EI-MS: *m/z* 408; Anal. calcd. for $C_{22}H_{20}N_2O_4S$: C; 64.69, H; 4.94, N; 6.86. Found: C; 64.54, H; 5.05, N; 6.97.

Dimethyl 5-[3,4-bis(octyloxy)benzoyl]benzene-1,3-dioate *p*-tolylsulfonylhydrazone (16f) Treatment as in the procedure of **16a** using **15f** (261 mg, 0.47 mmol) and *p*-tosylhydrazide (263 mg, 1.41 mmol) gave **16f** (253 mg, 75%). ^{1}H NMR ($CDCl_3$, 200 MHz): δ 8.80 (s, 1H), 8.67 (d, *J* = 13.8 Hz, 1H), 8.24 (s, 1H), 8.01–7.76 (m, 4H), 7.37–7.20 (d, *J* = 8.4 Hz, 2H), 6.98 (d, *J* = 8.0 Hz, 1H), 6.7–6.55 (m, 2H), 4.11–3.86 (m, 10H), 2.43 (s, 3H), 1.89–1.72 (m, 4H), 1.30–1.21 (m, 20H), 0.89 (t, *J* = 4.0 Hz, 6H); ^{13}C NMR ($CDCl_3$, 50.3 MHz): δ 165.23, 164.79, 164.67, 150.06, 150.85, 150.37, 149.59, 148.50, 143.84, 143.71, 137.74, 134.99, 134.93, 134.11, 133.36, 132.32, 131.56, 131.06, 130.79, 130.38, 129.32, 129.21, 128.37, 127.67, 121.54, 120.96, 113.62, 112.71, 111.89, 111.40, 69.22, 68.99, 52.51, 52.36, 31.82, 31.79, 29.31, 29.26, 29.19, 26.08, 26.01, 22.69, 21.67, 14.15; FT-IR (KBr, cm^{-1}): 3182, 2952, 2925, 2855, 1731, 1598, 1511, 1430, 1343, 1246; EI-MS: *m/z* 722; Anal. calcd. for $C_{40}H_{54}N_2O_8S$: C; 66.46, H; 7.53, N; 3.87. Found: C; 66.22, H; 7.21, N; 3.20.

4-{1-(2-Tosylhydrazono)-1-[3,4-bis(octyloxy)phenyl]methyl}benzonitrile (16g) Treatment as in the procedure of **16a** using **15g** (2.87 g, 6.12 mmol) and *p*-toluenesulfonylhydrazine (3.4 g, 18.5 mmol) gave **16g** (2.76 g, 71%). ^{1}H NMR ($CDCl_3$, 200 MHz): δ 8.10 (s, 1H), 7.85 (d, *J* = 8.2 Hz, 2H), 7.54 (m, 4H) 7.34 (d, *J* = 8.0 Hz, 2H), 6.98 (d, *J* = 8.2 Hz, 1H), 6.66 (d, *J* = 8.2 Hz, 1H), 6.56 (d, *J* = 2.0 Hz, 1H), 4.04–3.85 (m, 4H), 2.41 (s, 3H), 1.83–1.74 (m, 4H), 1.48–1.29 (m, 20H), 0.88 (t, *J* =3.0 Hz, 6H); ^{13}C NMR ($CDCl_3$, 50.3 MHz): δ 151.50, 150.14, 149.49, 143.61, 140.43, 134.94, 131.35, 129.17, 127.50, 127.31, 121.19, 120.64, 117.93, 113.52, 112.50, 112.27, 69.00, 68.75, 31.63, 31.61, 29.18, 29.12, 29.08, 29.00, 25.89, 25.84, 22.52, 21.43, 13.99; FT-IR (KBr, cm^{-1}): 3151, 2923, 2855, 1599, 1514, 1472, 1405, 1346,

1305; Anal. calcd. for $C_{37}H_{49}N_3O_4S$: C; 70.33, H; 7.82, N; 6.65. Found: C; 70.07, H; 7.74, N; 6.53.

1-(4-Nitrophenyl)-1-[3,4-bis(octyloxy)phenyl]methanone *p*-tolylsulfonylhydrazone (16h)

Treatment as in the procedure of **16a** using **15h** (3.00 g, 6.20 mmol) and *p*-tosylhydrazine (3.40 g, 18.6 mmol) gave **16h** (2.78 g, 69%) of the title compound. ^{1}H NMR ($CDCl_3$, 200 MHz): δ 8.12 (m, 3H), 7.86 (d, *J* = 8.2 Hz, 2H), 7.64 (m, 2H), 7.36 (d, *J* = 8.4 Hz, 1H), 7.00 (d, *J* = 8.2 Hz, 1H), 6.68 (d, *J* = 9.8 Hz, 1H), 6.57 (d, *J* = 2.0 Hz, 1H), 4.07 (m, 4H), 2.43 (s, 3H), 1.89–1.73 (m, 4H), 1.49–1.20 (m, 20H), 0.89 (t, *J* = 6.8 Hz, 6H); ^{13}C NMR ($CDCl_3$, 50.3 MHz): δ 151.26, 150.43, 149.79, 147.76, 143.98, 142.31, 135.03, 129.40, 127.94, 127.53, 123.02, 121.23, 120.73, 113.70, 112.58, 69.27, 69.03, 31.84, 31.81, 31.58, 29.37, 29.31, 29.27, 29.18, 26.08, 26.02, 22.71, 21.66, 14.18; FT-IR (KBr, cm^{-1}): 2925, 2856, 1598, 1517, 1423, 1348, 1254, 1170; EI-MS *m*/*z* 651; Anal. calcd. for $C_{36}H_{49}N_3O_6S$: C; 66.33, H; 7.58, N; 6.45. Found: C; 67.03, H; 7.53, N; 6.30.

1-(3-Nitrophenyl)-1-[3,4-bis(octyloxy)phenyl]methanone *p*-tolylsulfonylhydrazone (16i)

Treatment as in the procedure of **16a** using **15i** (2.00 g, 4.31 mmol) and *p*-tosylhydrazine (2.4 g, 12.94 mmol) gave **16i** (2.4 g, 88%). ^{1}H NMR ($CDCl_3$, 200 MHz): δ 8.36 (dd, *J* = 1.2, 8.2 Hz, 1H), 7.95 (t, *J* = 1.6 Hz, 1H), 7.84 (d, *J* = 8.4 Hz, 1H), 7.75 (m, 2H) 7.56 (d, *J* = 7.8 Hz, 1H) 7.34–7.19 (m, 3H), 6.69 (d, *J* = 8.6 Hz, 1H), 6.54 (d, *J* = 2.2 Hz, 1H), 3.99–3.93 (m, 4H), 2.44 (s, 3H), 1.86–1.75 (m, 4H), 1.47–1.29 (m, 20H), 0.89 (t, *J* = 6.4 Hz, 6H); ^{13}C NMR ($CDCl_3$, 50.3 MHz): δ 151.70, 151.13, 148.68, 148.45, 144.11, 134.79, 134.44, 132.98, 130.56, 129.43, 128.09, 127.72, 124.55, 123.44, 121.63, 111.97, 111.44, 69.20, 69.01, 31.95, 31.90, 29.53, 29.43, 29.35, 29.19, 29.21, 26.09, n22.82, 22.79, 21.79, 14.26; FT-IR (KBr, cm^{-1}): 3903, 3838, 3736, 1726, 1647, 1599, 1569; EI-MS: *m/z* 651; Anal. calcd. for $C_{36}H_{49}N_3O_6S$: C; 66.33, H; 7.58, N; 6.45. Found: C; 66.03, H; 7.48, N; 6.43.

1-(4-Methylsulfonylphenyl)-1-[3,4-bis(octyloxy)phenyl]methanone *p*-tolylsulfonylhydrazone (16j)

Treatment as in the procedure of **16a** using **15j** (1.40 g, 2.70 mmol) and *p*-tosylhydrazine (1.51 g, 8.10 mmol) gave **16j** (1.80 g, 97%). ^{1}H NMR ($CDCl_3$, 200 MHz): δ 0.91–0.85 (m, 6H), 1.61–

1.20 (m, 20H), 1.98–1.75 (m, 4H), 2.44 (s, 3H), 3.03 (s, 3H), 4.13–3.85 (m, 4H), 6.66–6.53 m, 2H), 6.99–6.94 (m, 1H), 7.35–7.31 (m, 2H), 7.65–7.60 (m, 2H), 7.86–7.82 (m, 5H); FT-IR (KBr, cm^{-1}): 3209, 3019, 2923.

1-[3,4-Bis(octyloxy)phenyl]-1-(4-trifluoromethylsulfonylphenyl)methanone *p*-tolylsulfonylhydrazone (16k)

Treatment as in the procedure of **16a** using **15k** (2.93 g, 5.14 mmol) and *p*-tosylhydrazine (2.87 g, 15.4 mmol) gave **16k** (1.80 g, 97%). ^{1}H NMR ($CDCl_3$, 200 MHz): δ 8.15–7.70 (6H, m), 7.50–7.17 (2H, m), 7.01–6.96 (1H, m), 6.67–6.44 (2H, m), 4.09–3.87 (4H, m), 2.45 (3H, s), 1.98–1.72 (4H, m), 1.77–1.15 (20H, m), 0.91–0.88 (6H, m); ^{19}F NMR ($CDCl_3$, 188 MHz,): δ 77.62, 77.72; FT-IR (KBr, cm^{-1}): 3568, 3211, 2928, 1598.

Methyl {[6,6]-1-[3,4,5-tris(octyloxy)phenyl]-C61}-4-benzoate (1b)

Treatment as in the procedure of **1a** using **16b** (737 mg, 0.929 mmol), sodium methoxide (50 mg, 0.929 mmol) and C_{60} (401 mg, 0.557 mmol) gave **1b** (163 mg, 22%). ^{1}H NMR ($CDCl_3$, 200 MHz): δ 8.32 (d, *J* = 8.4 Hz, 2H), 8.13 (d, *J* = 8.4 Hz, 2H), 7.63 (d, *J* = 8.6 Hz, 1H), 6.56 (d, *J* = 5.6 Hz, 1H), 4.21–3.95 (m, 6H), 3.91 (s, 3H), 1.85–1.59 (m, 6H), 1.45–1.27 (m, 30H), 0.87 (t, *J* = 6.6 Hz, 9H); ^{13}C NMR ($CDCl_3$, 50.3 MHz): δ 166.42, 151.52, 149.54, 148.18, 148.03, 147.72, 145.49, 145.19, 144.96, 144.85, 144.36, 144.27, 143.94, 143.81, 143.60, 143.49, 143.15, 142.62, 142.19, 142.09, 141.88, 141.67, 140.62, 140.35, 138.25, 137.80, 137.60, 136.36, 134.62, 131.18, 129.63, 129.48, 129.30, 126.42, 126.24, 115.82, 109.41, 104.75, 101.28, 78.65, 73.50 (bridgehead), 72.97, 70.39, 68.61, 52.73 (bridge), 52.23, 31.96, 30.36, 30.17, 29.43, 26.38, 26.19, 26.02, 22.85, 14.32; FT-IR (KBr, cm^{-1}): 2922, 2851, 1725, 1609, 1505, 1455, 1431, 1274, 1187, 1098, 1020; MALDI-TOF MS: *m/z* 1328; Anal. calcd. for $C_{99}H_{60}O_5$: C; 89.43 H; 4.55. Found: C; 89.08, H; 3.94.

Methyl {[6,6]-1-[4-(octyloxy)phenyl]-C61}-4-benzoate (1c)

Treatment as in the procedure of **1a** using **16c** (1.3 g, 2.42 mmol), sodium methoxide (131 mg, 2.42 mmol) and C_{60} (1.00 g, 1.45 mmol) gave **1c** (317 mg, 21%). ^{1}H NMR ($CDCl_3$, 200 MHz): δ 8.13 (m, 4H), 8.0 (d, *J* = 8.6 Hz, 2H), 6.99 (d, *J* = 8.6 Hz, 2H), 3.99 (t, *J* = 6.4 Hz, 2H), 3.91 (s, 3H), 1.81–1.74 (m, 2H), 1.29–1.21 (m, 10H), 0.91 (t, *J* = 6.2 Hz, 3H); ^{13}C NMR ($CDCl_3$, 50.3

MHz): δ 166.15, 158.65, 147.57, 144.95, 144.84, 144.76, 144.35, 144.29, 144.21, 144.0, 143.96, 143.47, 142.65, 142.61, 141.93, 141.85, 141.79, 140.56, 138.00, 137.65, 131.79, 130.52, 129.89, 129.68, 129.48, 114.62, 78.80, 68.06, 57.16, 52.27, 31.94, 29.52, 29.43, 29.39, 26.27, 22.83, 14.34; FT-IR (KBr, cm^{-1}): 2923, 2853, 1725, 1608, 1511, 1464, 1430, 1275, 1186, 1136, 1107; MALDI-TOF MS: *m/z* 1072; Anal. calcd. for $C_{83}H_{28}O_3$: C; 92.90, H; 2.63. Found: C; 92.17, H; 2.59.

Methyl {[6,6]-1-(3,4-dimethoxyphenyl)-C61}-4-benzoate (1d)

Treatment as in the procedure of **1a** using **16d** (259 mg, 0.55 mmol), sodium methoxide (30 mg, 0.55 mmol) and C_{60} (239 mg, 0.33 mmol) gave **1d** (112 mg, 34%). ^{1}H NMR ($CDCl_3$, 200 MHz): δ 8.15 (m, 4H), 7.67 (dd, *J* = 2.2, 8.4 Hz, 1H), 7.53 (d, *J* = 2.0 Hz, 1H), 6.99 (d, *J* = 8.2 Hz, 1H), 3.97 (d, *J* = 9.0 Hz, 9H); ^{13}C NMR ($CDCl_3$, 50.3 MHz): δ 166.19, 148.78, 148.63, 147.46, 144.95, 144.80, 144.42, 144.38, 144.34, 144.23, 144.00, 143.85, 143.50, 142.72, 142.63, 142.59, 141.94, 141.87, 141.80, 140.62, 140.58, 138.12, 137.70, 130.59, 130.30, 129.88, 129.60, 123.76, 113.84, 111.09, 78.72, 57. 39, 56.46, 55.96, 52.32; FT-IR (KBr, cm^{-1}): 1721, 1607, 1511, 1460, 1430, 1407, 1274, 1249; MALDI-TOF MS: *m/z* 1004; Anal. calcd. for $C_{77}H_{16}O_4$: C; 92.03, H; 1.60, Found: C; 91.13, H; 1.59.

Methyl {[6,6]-1-phenyl-C61}-4-benzoate (1e)

Treatment as in the procedure of **1a** using **16e** (700 mg, 1.71 mmol), sodium methoxide (93.0 mg, 1.71 mmol) and C_{60} (741 mg, 1.03 mmol) yielded **1e** (335 mg, 35%). ^{1}H NMR ($CDCl_3$, 200 MHz): δ 8.16–8.02 (m, 4H), 7.79 (m, 1H), 7.52–7.30 (m, 2H), 7.21–7.06 (m, 2H), 3.91 (s, 3H); ^{13}C NMR ($CDCl_3$, 50.3 MHz): δ 165.42, 165.18, 147.13, 147.08, 144.69, 144.62, 144.19, 144.14, 144.06, 143.84, 143.73, 143.30, 143.17, 142.46, 141.62, 141.23, 140.46, 140.27, 137.81, 137.36, 137.60, 136.01, 134.45, 133.40, 132.32, 130.55, 130.47, 120.71, 129.64, 129.51, 129.29, 129.10, 129.06, 128.83, 128.54, 128.05, 128.01, 127.20, 126.94, 124.57, 78.17, 57.36, 52.05, 51.89, 29.93, 21.74, 21.58; FT-IR (KBr, cm^{-1}): 1725, 1608, 1432; MALDI-TOF MS: *m/z* 944; Anal. calcd. for $C_{75}H_{12}O_2$: C;95.33, H; 1.28. Found: C;84.87, H; 1.23.

Dimethyl {[6,6]-1-[3,4-bis(octyloxy)phenyl]-C61}benzen-3,5-dioate (1f)

Treatment as in the procedure of **1a** using **16f** (218 mg, 0.30 mmol), sodium methoxide (16.0 mg, 0.30 mmol) and C_{60} (131 mg, 0.18 mmol)gave **1f** (89 mg, 39%). ^{1}H NMR ($CDCl_3$, 200 MHz): δ 8.93 (m, 2H), 8.69 (d, *J* = 1.6 Hz, 1H), 7.67 (dd, *J* = 2.2, 8.2 Hz, 1H), 7.56 (d, *J* = 2.2 Hz, 1H), 6.96 (d, *J* = 8.2 Hz, 1H), 4.13–3.98 (m, 4H), 3.97 (s, 6H), 1.85–1.76 (m, 4H), 1.46–1.28 (m, 20H), 0.89 (t, *J* = 5.4 Hz, 6H); ^{13}C NMR ($CDCl_3$, 50.3 MHz): δ 165.42, 149.27, 148.39, 147.43, 147.14, 145.15, 144.92, 144.88, 144.84, 144.80, 144.46, 144.36, 144.18, 144.10, 144.02, 143.54, 143.47, 142.74, 142.69, 142.65, 142.59, 141.92, 141.84, 140.66, 140.63, 140.37, 138.16, 137.64, 135.55, 130.93, 130.09, 129.87, 123.99, 117.02, 113.20, 78.69, 69.83, 69.02, 56.72, 52.67, 32.00, 31.96, 29.65, 29.54, 29.45, 26.27, 26.22, 22.87, 22.86, 14.36; FT-IR (KBr, cm^{-1}): 2923, 2853, 1730, 1717, 1508, 1427; MALDI-TOF MS: *m/z* 1258; Anal. calcd. for $C_{93}H_{46}O_6$: C; 88.70, H; 3.68. Found: C; 87.97, H; 3.65.

4-{[6,6]-1-[3,4-Bis(octyloxy)phenyl]-C61}benzonitrile (1g)

Treatment as in the procedure of **1a** using **16g** (1.16 g, 1.83 mmol), sodium methoxide (99 mg, 1.83 mmol) and C_{60} (794 mg, 1.1 mmol) of in ODCB (15 mL) gave **1g** (204 mg, 19%). ^{1}H NMR ($CDCl_3$, 200 MHz): δ 8.23 (d, *J* = 9.0 Hz, 2H), 7.77 (d, *J* = 8.2 Hz, 2H), 7.59(m, 2H), 6.96 (d, *J* = 8.2 Hz, 1H), 4.11–3.97 (m, 4H), 1.86–1.73 (m, 4H), 1.46–1.28 (m, 20H), 0.88 (t, *J* = 7.4 Hz, 6H); ^{13}C NMR ($CDCl_3$, 50.3 MHz): δ 149.39, 148.41, 147.18, 147.03, 144.87, 144.84, 144.81, 144.65, 144.42, 144.33, 144.27, 144.12, 144.09, 144.02, 143.97, 143.47, 143.43, 142.64, 142.61, 142.52, 141.88, 141.77, 140.60, 138.15, 137.55, 132.36, 131.21, 129.42, 128.76, 127.95, 125.03, 123.93, 118.27, 117.01, 113.11, 111.76, 78.40, 69.84, 68.97, 56.93, 31.96, 31.92, 29.62, 29.50, 29.42, 26.24, 26.20, 22.85, 22.82, 14.35; FT-IR (KBr, cm^{-1}): 2921, 2852, 1508, 1457, 1264, 1249; MALDI-TOF MS: *m/z* 1167; Anal. calcd. For $C_{90}H_{41}NO_2$: C; 92.52, H; 3.54, N; 1.20. Found: C; 91.37, H; 3.54, N; 1.11.

[6,6]-1-(4-Nitrophenyl) -1-[3,4-bis(octyloxy)phenyl]-C61 (1h)

Treatment as in the procedure of **1a** using **16h** (1.48 g, 2.28 mmol), sodium methoxide (123 mg, 2.28 mmol) and C_{60} (986 mg, 1.36 mmol) gave **1h** (344 mg, 26%). ^{1}H NMR ($CDCl_3$, 200 MHz): δ 8.36 (m, 2H), 8.13 (d, *J*=8.6 Hz, 1H), 7.62 (m, 2H), 7.32 (d, *J* = 8.6 Hz, 1H), 6.98 (d, *J* = 8.2 Hz, 1H), 4.12–3.89 (m, 4H), 1.86–1.74 (m, 4H), 1.56–1.25 (m, 20H), 0.95 (t, *J* = 6.8 Hz, 6H); ^{13}C

NMR ($CDCl_3$, 50.3 MHz): δ 149.53, 149.09, 149.00, 148.53, 147.82, 147.13, 146.96, 146.08, 144.90, 144.68, 144.50, 144.42, 144.32, 144.12, 143.54, 142.73, 142.61, 141.93, 141.82, 140.70, 138.24, 137.62, 131.49, 131.43, 130.64, 129.27, 128.57, 124.05, 123.94, 123.49, 121.11, 117.13, 114.78, 114.00, 113.23, 78.41, 69.93, 69.40, 69.05, 68.16, 56.60, 41.38, 38.86, 31.96, 30.52, 29.85, 29.65, 29.54, 29.43, 29.08, 26.22, 23.94, 23.17, 22.85, 14.32; FT-IR (KBr, cm^{-1}): 2924, 2854, 1654, 1596, 1514; MALDI-TOF MS: *m/z* 1187; Anal. calcd. for $C_{89}H_{41}NO_4$: C; 89.96, H; 3.48, N; 1.18. Found: C; 89.13, H; 3.43, N; 1.16.

[6,6]-1-(3-Nitrophenyl) -1-[3,4-bis(octyloxy)phenyl]-C61 (1i)

Treatment as in the procedure of **1a** using **16i** (1.79 g, 2.83 mmol), sodium methoxide and C_{60} (1.22 g, 1.7 mmol) yielded **1i** (640 mg, 32%). ^{1}H NMR ($CDCl_3$, 200 MHz): δ 8.93 (s, 1H), 8.46 (d, *J* = 9.2 Hz, 1H), 8.26 (d, *J* = 8.2 Hz, 1H) 7.70–7.53 (m, 2H), 6.98 (d, *J* = 8.2 Hz, 1H), 6.81 (s, 1H), 4.13–3.98 (m, 4H), 1.83–1.79 (m, 4H), 1.43–1.28 (m, 20H), 0.87 (br s, 6H); ^{13}C NMR ($CDCl_3$, 50.3 MHz): δ 149.43, 148.50, 147.95, 147.17, 146.90, 144.92, 144.69, 144.49, 144.39, 144.13, 144.05, 143.54, 143.48, 142.72, 142.59, 141.91, 141.82, 141.18, 140.71, 138.20, 137.62, 136.51, 129.56, 125.35, 123.94, 122.97, 116.97, 113.21, 78.45, 69.88, 69.33, 69.04, 56.42, 31.96, 29.85, 29.64, 29.43, 26.21, 22.85, 14.31; FT-IR (KBr, cm^{-1}): 2923, 2854, 1588, 1528, 1510, 1463; MALDI-TOF MS: *m/z* 1187; Anal. calcd. for $C_{89}H_{41}NO_4$: C; 89.96, H; 3.48, N; 1.18. Found: C; 89.13, H; 3.43, N; 1.16.

[6,6]-1-(4-Methylsulfonylphenyl)-1-[3,4-bis(octyloxy)phenyl]-C61 (1j)

Treatment as in the procedure of **1a** using **16j** (1.80 g, 2.62 mmol), sodium methoxide (212 mg, 3.93 mmol) and C_{60} (1.13 g, 2.62 mmol) gave **1j** (422 mg, 22%). ^{1}H NMR ($CDCl_3$, 200 MHz): δ 0.91–0.84 (m, 6H), 1.53–1.29 (m, 20H), 1.90–1.75 (m, 4H), 3.15 (s, 3H), 4.12–3.98 (m, 4H), 6.96 (d, *J* = 8.4 Hz, 1H), 7.62–7.57 (m, 2H), 8.04 (d, *J* = 8.0 Hz, 2H), 8.30 (d, *J* = 8.0 Hz, 2H); ^{13}C NMR ($CDCl_3$, 50.3 MHz): δ 14.32, 14.36, 22.86, 26.22, 26.26, 29.45, 29.53, 29.63, 31.94, 31.96, 44.59, 56.78, 56.78, 69.03, 69.91, 78.41, 113.13, 117.13, 124.00, 127.72, 129.55, 131.45, 137.72, 138.19, 139.72, 140.66, 141.83, 141.91, 142.61, 142.68, 143.52, 144.04, 144.09, 144.18, 144.31, 144.40, 144.47, 144.71, 144.87, 144.91, 145.11, 146.98, 147.23, 148.46, 149.42. FT-IR (KBr, cm^{-1}): 3423, 2923, 2852, 1594, 1509, 1464, 1425, 1319, 1151, 1090, 1019; MALDI-TOF MS: *m*/z 1220.

[6,6]-1-[3,4-bis(octyloxy)phenyl]-1-(4-trifluoromethylsulfonylphenyl)-C61 (1k)

To a solution of **16k** (1.46 g, 1.97 mmol) in ODCB (2 mL) was added lithium hexamethyldisilazide (0.64 M THF solution, 1.84 mL, 1.18 mmol) and stirred for 0.5 h at room temperature. A solution of C_{60} (851 mg, 1.18 mmol) in ODCB (14 mL) was added and the reaction mixture was heated for 24 h at 80 ˚C. The reaction mixture was washed with diluted HCl aqueous solution and brine successively. Solvents were removed under vacuum to give the crude product which was purified by column chromatography (silica gel; toluene). First fraction was unreacted $C_{60.}$ Fractions containing brown color were collected and the solvent was evaporated to leave a solid. This compound was dissolved in toluene and refluxed for 24 h to complete the isomerisation. The isomerisation was confirmed by HPLC (Develosil ODS-HG-5, MeOH/$CHCl_3$) and ^{13}C NMR. Further purification by column chromatography as above yielded **1k** (292 mg, 19%). ^1H NMR ($CDCl_3$, 200 MHz): δ 8.15–7.70 (m, 6H), 7.50–7.17 (m, 2H), 7.01–6.96 (m, 1H), 6.67–6.44 (m, 2H), 4.09–3.87 (m, 4H), 2.45 (s, 3H), 1.98–1.72 (m, 4H), 1.77–1.15 (m, 20H), 0.91–0.88 (m, 6H); ^{13}C NMR ($CDCl_3$, 50.3 MHz): δ 14.12, 22.64, 22.66, 26.02, 26.08, 26.88, 29.24, 29.27, 29.32, 29.35, 29.46, 31.55, 31.78, 31.81, 56.41, 68.95, 69.87, 76.79, 77.00, 77.21, 78.11, 113.23, 117.25, 118.67, 120.82, 124.33, 129.09, 130.67, 131.23, 132.14, 137.82, 138.50, 140.94, 140.99, 142.06, 142.09, 142.14, 142.86, 142.94, 142.98, 143.00, 143.03, 143.75, 143.79, 144.34, 144.40, 144.56, 144.68, 144.76, 144.87, 145.10, 145.17, 145.20, 145.23, 146.92, 147.18, 148. 49, 148.78, 149.91; FT-IR (KBr, cm^{-1}): 3446, 2924, 2853.17, 1593, 1509. MALDI-TOF MS: *m/z* 1274.

Methyl (4-{1-[3,4-bis(octyloxy)phenyl}-C71)benzoate (2)

Treatment as in the procedure of **1a** using **16a** (650 mg, 0.977 mmol), sodium methoxide (52.8 mg, 0.977 mmol) and C_{70} (493 mg, 0.586 mmol) gave **2** (245 mg, 32%). ^1H NMR ($CDCl_3$, 200 MHz): δ 8.16 (m, 4H), 7.6 (dd, J = 2.0, 8.2 Hz, 1H), 7.35 (br, s, 1H), 6.92 (d, J = 8.4 Hz, 1H), 4.13–3.94 (m, 4H), 3.89 (s, 3H), 1.80–1.78 (m, 4H), 1.60–1.27 (m, 20H), 0.87 (t, J = 4.2 Hz, 6H); ^{13}C NMR ($CDCl_3$, 50.3 MHz): δ 166.10, 155.19, 155.01, 151.61, 151.06, 150.80, 150.51, 150.14, 149.33, 149.08, 148.79, 148.34, 148.14, 147.92, 147.61, 147.13, 146.60, 145.98, 145.61, 145.39, 144.76, 144.27, 144.14, 143.59, 143.42, 143.01, 142.29, 141.47, 141.21, 139.87, 139.59, 138.07, 133.67, 132.51,130.93, 130.51, 130.28, 130.14, 129.45, 128.67, 128.34, 123.44, 116.50,

113.13, 71.48, 69.82 (bridgehead), 69.58, 68.97, 52.30, 41.32 (bridge), 32.01, 31.72, 29.70, 29.43, 26.27, 22.86, 14.34; FT-IR (KBr, cm^{-1}): 2919, 2850, 1725; MALDI-TOF MS: *m/z* 1320.
